# Feasibility of dose calculation for treatment plans using electron density maps from a novel dual-layer detector spectral CT simulator

**DOI:** 10.1186/s13014-024-02479-6

**Published:** 2024-07-24

**Authors:** Qizhen Zhu, Shuoyang Wei, Zhiqun Wang, Haoran Xu, Bing Zhou, Huiying Qu, Mingming Nie, Ning Guo, Wenshuai Wang, Bo Yang, Jie Qiu

**Affiliations:** 1 Department of Radiation Oncology, Peking Union Medical College Hospital, Chinese Academy of Medical Sciences and Peking Union Medical College, Beijing, China; 2 Philips Clinical Science, Beijing, China

**Keywords:** Spectral CT simulator, Electronic density, Dose calculation, Dosimetry comparison

## Abstract

**Background:**

Conventional single-energy CT can only provide a raw estimation of electron density (ED) for dose calculation by developing a calibration curve that simply maps the HU values to ED values through their correlations. Spectral CT, also known as dual-energy CT (DECT) or multi-energy CT, can generate a series of quantitative maps, such as ED maps. Using spectral CT for radiotherapy simulations can directly acquire ED information without developing specific calibration curves. The purpose of this study is to assess the feasibility of utilizing electron density (ED) maps generated by a novel dual-layer detector spectral CT simulator for dose calculation in radiotherapy treatment plans.

**Methods:**

30 patients from head&neck, chest, and pelvic treatment sites were selected retrospectively, and all of them underwent spectral CT simulation. Treatment plans based on conventional CT images were transplanted to ED maps with the same structure set, including planning target volume (PTV) and organs at risk (OARs), and the dose distributions were then recalculated. The differences in dose and volume histogram (DVH) parameters of the PTV and OARs between the two types of plans were analyzed and compared. Besides, gamma analysis between these plans was performed by using MEPHYSTO Navigator software.

**Results:**

In terms of PTV, the homogeneity index (HI), gradient index (GI), D_2%_, D_98%_, and D_mean_ showed no significant difference between conventional plans and ED plans. For OARs, statistically significant differences were observed in parotids D_50%_, brainstem in head&neck plans, spinal cord in chest plans and rectum D_50%_ in pelvic plans, whereas the variance remained minor. For the rest, the DVH parameters exhibited no significant difference between conventional plans and ED plans. All of the mean gamma passing rates (GPRs) of gamma analysis were higher than 90%.

**Conclusion:**

Compared to conventional treatment plans relying on CT images, plans utilizing ED maps demonstrated similar dosimetric quality. However, the latter approach enables direct utilization in dose calculation without the requirements of establishing and selecting a specific Hounsfield unit (HU) to ED calibration curve, providing an advantage in clinical applications.

## Introduction

Computed tomography (CT) has been used in the field of radiotherapy for decades, and now, most radiation oncology departments have a dedicated CT system used as a simulator for radiotherapy. However, conventional CT imaging may not always provide optimal soft tissue contrast, making it difficult to distinguish between different types of soft tissues [1, 2]. This can be particularly challenging when targeting tumors surrounded by or adjacent to critical organs or normal tissues. Besides, conventional single-energy CT can only provide a raw estimation of electron density (ED) for dose calculation by developing a calibration curve that simply maps the HU values to ED values through their correlations [3, 4]. To prevent subsequent errors in dose calculation, the development of calibration curves requires medical physicists to perform the task properly with specific calibration settings. Hence, it is not always possible to produce the best image quality for individual patients due to concerns about deviations from the calibration settings, which frequently limit the patient scans to predetermined parameters (e.g., the tube potential, radiation exposure, or reconstruction filter).

Spectral CT, also known as dual-energy CT (DECT) or multi-energy CT, was clinically introduced for the diagnostic imaging field in 2006 [5]. By acquiring two energy-level X-ray data and using specific decomposition algorithms, spectral CT systems can generate a quantitative dataset [6, 7]. Through the datasets, several quantitative maps can be reconstructed, such as virtual monochromatic images (VMI) representing mono-energetic photon energies at different kilo electron Volt (keV) levels, single material decompositions, virtual non-contrast images (VNC), ED maps and effective atomic numbers (Z_eff_) maps [8]. Recently, a novel spectral CT scanner has been introduced into clinical use, which has the unique property of creating spectral separation at the detector level. Philips Spectral CT 7500 (Philips Healthcare, Best, The Netherlands) features a novel dual-layer detector, which can simultaneously acquire high- and low-energy X-ray data. The configuration of the dual-layer detector consists of two layers of scintillators made from different materials: the top layer, based on yttrium, primarily absorbs low-energy photons from the x-ray beam, while the bottom layer, made of gadolinium oxysulphide (GOS), absorbs higher-energy photons that are transmitted and hardened by the top layer [9–12]. Understanding the quantitative accuracy of the ED and Zeff is a critical first step toward making such a paradigm shift in radiation therapy planning. Some phantom studies have been conducted to evaluate the accuracy of determining ED values in ED maps for dual-layer spectral CT systems [8, 13], And the results show the high validity of ED value estimation based on spectral CT. For dose calculation, Atez et al. [14]. investigated whether using ED maps could reduce the calculation error caused by the iodine contrast agent compared with using conventional CT images in post-contrast scans. Their results showed that the dose distribution calculated based on ED maps is more similar to that based on unenhanced CT.

However, there is still a lack of research on the clinical feasibility of using ED maps for photon dose calculation and directly comparing the difference between dose distributions of treatment plans based on ED maps and conventional CT images. This study aimed first to quantify the accuracy of the determination of relative ED values using clinical simulation scanning protocols with a new dual-layer spectral CT system. Second, to explore the feasibility of treatment plan dose calculation using the ED maps generated from real patients’ spectral data.

## Methods and materials

### Phantom configuration

**Fig. 1:**
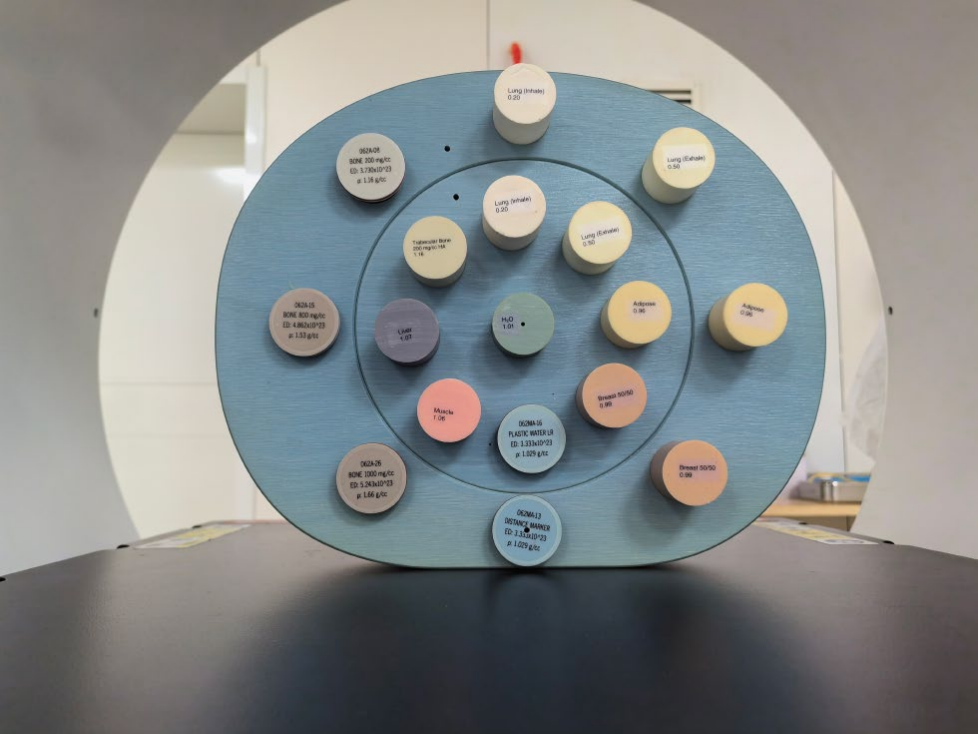
Insert arrangements of 062 M phantom for measurements of the accuracy of the relative ED value

The 062M electronic density phantom (CIRS, Norfolk, VA, USA) with various tissue-equivalent inserts was used to test the accuracy of the relative ED value of the ED map. It should be declared that the relative ED value is defined as the percentage of the ED of a substance relative to the ED of water (3.343 × 10^23^ m^− 3^). The expected relative ED values of these inserts were provided by the phantom vendor. The detailed information and arrangements for these inserts are shown in Table 1; Fig. 1.

**Table 1:** The detailed information of inserts of 062M phantom

Insert name	Physical density (g/cc)	Expected relative ED (%)
Lung inhale	0.21	19.00
Lung exhale	0.51	48.90
Adipose	0.96	94.90
Breast	0.99	97.60
Plastic water	1.00	100.00
Distance marker	1.03	105.20
Muscle	1.06	104.30
Trabecular Bone	1.16	111.70
Bone 200	1.16	111.70
Bone 800	1.53	145.60
Bone 1000	1.66	157.00

### Scanning and measurement

The specific scanning parameters for these four default scanning protocols utilized in clinical practice are as follows. The scan parameters for the head & neck protocol were 120 kVp, 350mAs, 55mGy CTDIvol, 0.5 pitch, 500-mm field of view (FOV), 64 × 0.625 mm collimation, and 3-mm slice thickness. For the chest protocol, the scan parameters were 120 kVp, 250 mAs, 19.4 mGy CTDIvol, 1 pitch, 128 × 0.625 mm collimation, 500-mm FOV, and 5-mm slice thickness. For the abdomen and pelvis protocol, the scan parameters were 120 kVp, 300 mAs, 23.2 mGy CTDIvol, 1 pitch, 128 × 0.625 mm collimation, 500-mm FOV, and 5 mm slice thickness. For the pediatrics protocol, the scan parameters were 100 kVp, 300 mAs, 15.4 mGy CTDIvol, 0.8 pitch, 64 × 0.625 mm collimation, 350 mm FOV, and 3 mm slice thickness.

All image series reconstructed conventional CT images and corresponding ED maps, and all of them were exported to Eclipse treatment planning system V15.6 (Varian Medical Systems, Palo Alto, CA).

Regions of interest (ROIs) were selected on the ED maps by using TPS’s contour module, which identifies the phantom center and orientation and performs data analysis at predefined locations. The diameter of the ROIs was 75% of the diameter of the cylindrical inserts. The measured values were compared with the corresponding expected values, including the absolute difference (measured—expected) and percentage deviation (100*[measured — expected]/expected).

### Treatment plans based on conventional CT images

In this study, the data of 30 patients from different treatment sites were selected retrospectively, including CT images and treatment plans. Among these conventional plans, 10 were head & neck plans, 10 were chest plans, and 10 were pelvic plans. The techniques used in the treatment plans include fixed-field intensity modulated radiation therapy (FF-IMRT) and volumetric modulated arc therapy (VMAT).

The prescribed dose for head & neck plans was 5040 cGy with 28 fractions. For chest plans, the prescribed dose was 4240 cGy with 16 fractions. For pelvic plans, the prescribed dose was 4500 cGy with 25 fractions. All selected treatment plans were designed and optimized by using the Eclipse TPS V15.6 and delivered by Halcyon2.0 and Truebeam linac. Anisotropic analytic algorithm (AAA) and phonon optimizer (PO) were used for dose calculation and plan optimization.

### Treatment plans based on ED maps and plan comparison

We transplanted the original treatment plans based on conventional CT images to ED maps with the same plan parameters and recalculated the dose distribution. The transplanted plans were referred to as ED plans. It should be noted that when transplanting the treatment plans, there is no need to register the two types of images beforehand because the ED maps and conventional CT images were homologous and acquired at the same time. In this study, we evaluated and compared the following DVH parameters of the planning target volume and OARs for both types of plans, respectively. For planning target volume (PTV), Paddick’s conformity index (CI), gradient index (GI) [15], and ICRU 83 homogeneity index (HI) [16] were defined as follows:

Conformity index (CI):

$$CI=\frac{{\text{(PTV volume receiving the prescription isodose)}}^{\text{2}}}{\text{(}\text{PTV volume * Prescription isodose volume)}}$$

represents the adequacy between the dose distribution and the shape of the target volume treated. The ideal value is 1.

Gradient index (GI):

$$GI=\frac{\text{Volume of the isodose } 50\% \text{ of the prescribed dose }}{\text{The volume of the isodose of the prescribed dose}}$$

represents the dose gradient between the prescribed dose level and 50% of the prescribed dose. The lowest possible value is ideal.

$$HI=\frac{{\text{D}}_{2\%}\text{-}{\text{D}}_{98\%}}{{\text{D}}_{50\%}}$$

corresponds to the homogeneity of the dose distribution of the target volume. The ideal value is 0.

For OARs of the head & neck treatment plans, the dose to the spinal cord ($({{\rm{D}}_{0.1c{m^3}}})$), parotids (D_mean_ and D_50%_), brain stem ($({{\rm{D}}_{0.1c{m^3}}})$) were scored. For the chest plans, both plans were evaluated the dose to the double lungs (V_20Gy_, D_mean_), lateral lung (D_mean_), contralateral lung (D_mean_), and heart (V_5Gy_ and D_mean_), and spinal cord ($({{\rm{D}}_{0.1c{m^3}}})$). For the pelvic plans, the dose to the small intestine (D_mean_, D_50%_ and ${\text{D}}_{{\text{2}\text{cm}}^{\text{3}}}$), Bladder (D_mean_, D_50%)_, Rectum (D_mean_, D_50%_), and spinal cord ($({{\rm{D}}_{0.1c{m^3}}})$) were recorded.

Besides, we exported these two kinds of treatment plans’ RT-Dose files in DICOM format and used MEPHYSTO Navigator software (PTW, Freiburg, Germany) for Gamma analyses. All gamma analyses were performed under absolute dose mode, and the gamma criteria were 1%/1 mm, 2%/2 mm, and 3%/2 mm with a 10% threshold of the maximum dose.

### Data analysis

Paired t-test was used to examine the significance of all DVH parameters’ differences via the SPSS 26, and a significance level of *P* < 0.05 was considered statistically significant.

## Results

### Phantom measurements

**Fig. 2:**
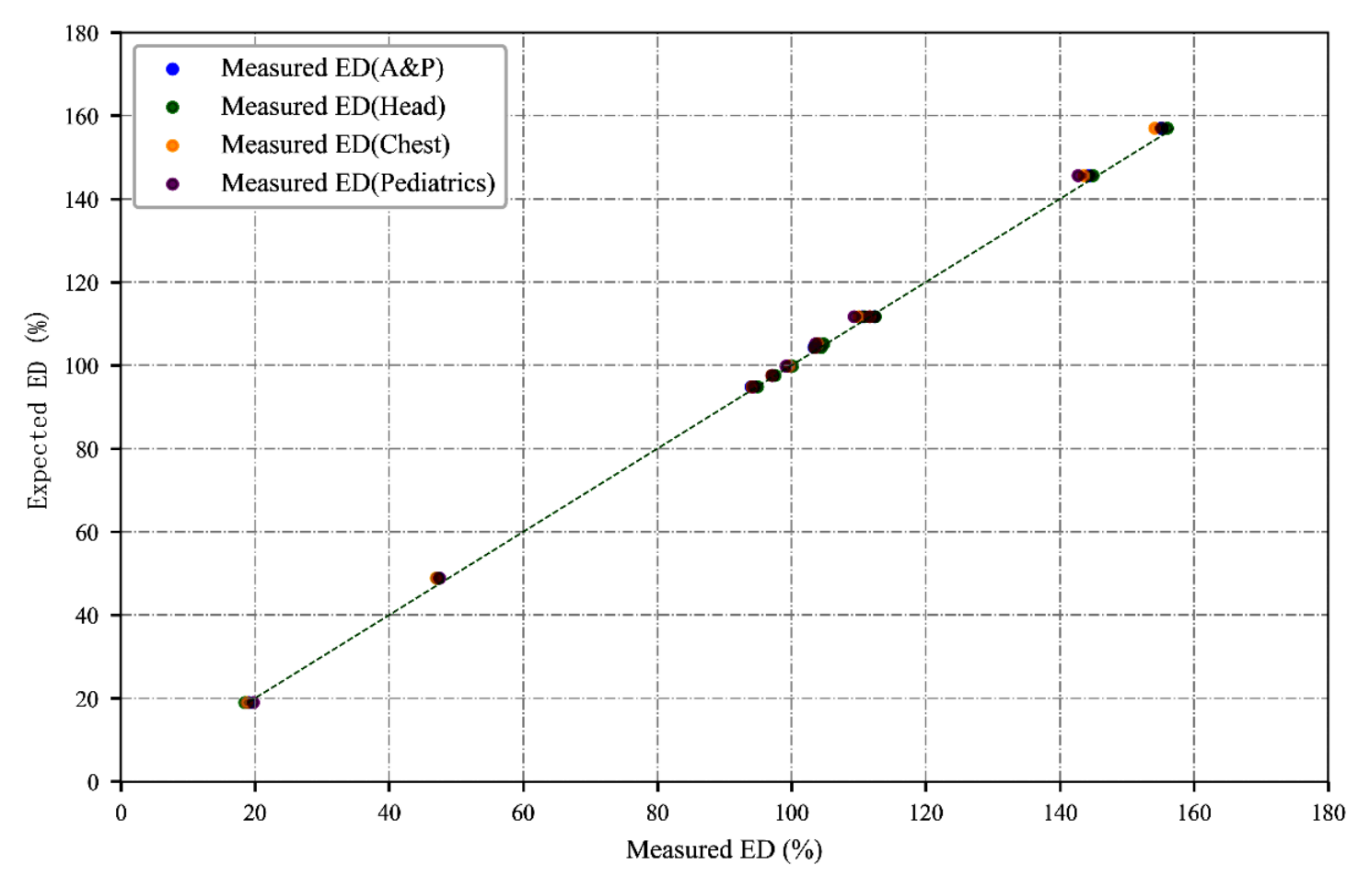
Accuracy of measured ED

Table 2; Fig. 2 show the accuracy of the measured ED values from the ED map.

Table 3 summarizes the percentage deviation of measured ED values compared with expected ED values. Slightly large percentage deviations were observed in the lung inserts (lung inhale and lunge exhale), and the maximum percentage deviation was less than 4%.

**Table 2:** Summary of the accuracy of the measured ED values

Name	Absolute error (%)			
	A&P	H&N	Chest	Pediatrics
Lung inhale	0.04	0.56	0.24	0.70
Lung exhale	1.68	1.81	1.93	1.42
Adipose	0.95	0.00	0.82	0.62
Breast	0.57	0.10	0.59	0.47
Plastic water	0.19	0.30	0.15	0.64
Distance marker	0.97	0.09	0.87	0.87
Muscle	1.24	0.44	1.31	1.68
Trabecular Bone	0.61	0.76	0.03	0.10
Bone 200	1.19	0.84	1.69	2.38
Bone 800	1.30	0.68	2.00	2.92
Bone 1000	2.00	1.02	2.90	1.80

**Table 3:** Summary of the percentage deviation of the measured ED values

Name	Percentage deviation (%)			
	A&P	H&N	Chest	Pediatrics
Lung inhale	0.21	2.95	1.26	3.68
Lung exhale	3.44	3.70	3.95	2.90
Adipose	1.00	0.00	0.86	0.65
Breast	0.58	0.10	0.60	0.48
Plastic water	0.19	0.30	0.15	0.64
Distance marker	0.93	0.09	0.83	0.83
Muscle	1.18	0.42	1.25	1.60
Trabecular Bone	0.55	0.68	0.03	0.09
Bone 200	1.07	0.75	1.51	2.13
Bone 800	0.89	0.47	1.37	2.01
Bone 1000	1.27	0.65	1.85	1.15

### Dosimetry comparison of PTV

**Fig. 3:**
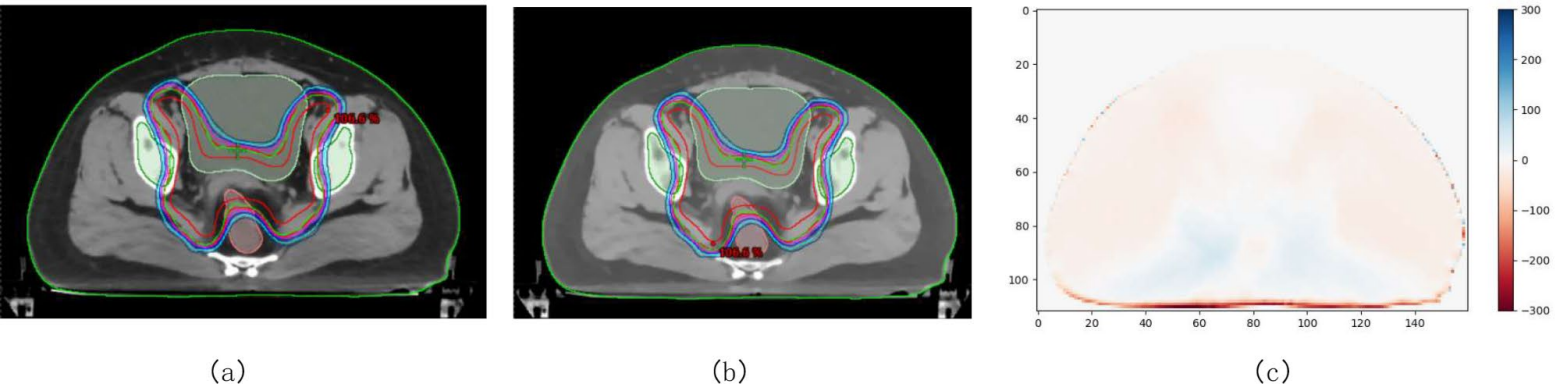
Photon dose distribution in an example pelvic patient (Patient 2). (**a**) Treatment plan based on the conventional CT image. (**b**) Treatment plan based on the ED map. (**c**) Difference map of dose distribution between the two treatment plans. ED, electron density

Table 4 summarizes the CI, HI, GI, D_2%_, D_98%_, and D_mean_ for both plans. For the plans in the head & neck treatment site, conventional plans were more conformal, shown by higher values of CI with statistical significance (see Table 4), but the difference was only 0.01. In general, similar dose distributions to PCTV were obtained for both plans. Figure 3. shows the dose distributions of an example patient (patient 2 in pelvic plans).

**Table 4:** DVH parameters comparison of conventional plans and ED plans for PTV(Mean ± SD)

DVH parameters	Conventional plans	ED plans	*P* value
**Head&Neck**			
CI	0.87 ± 0.02	0.86 ± 0.02	0.005
GI	3.81 ± 0.47	3.79 ± 0.45	0.249
HI	0.07 ± 0.01	0.08 ± 0.01	0.081
D_2%_(Gy)	53.40 ± 0.23	54.10 ± 1.87	0.240
D_98%_(Gy)	49.58 ± 0.11	49.52 ± 0.18	0.054
D_mean_(Gy)	52.20 ± 0.13	52.26 ± 0.19	0.125
**Chest**			
CI	0.82 ± 0.03	0.84 ± 0.04	0.276
GI	2.06 ± 0.14	2.09 ± 0.12	0.062
HI	0.08 ± 0.00	0.08 ± 0.00	0.168
D_2%_(Gy)	45.19 ± 0.31	45.07 ± 0.53	0.516
D_98%_(Gy)	41.60 ± 0.13	41.61 ± 0.13	0.841
D_mean_(Gy)	44.05 ± 0.19	44.03 ± 0.23	0.699
**Pelvis**			
CI	0.92 ± 0.01	0.93 ± 0.01	0.168
GI	3.73 ± 0.26	3.71 ± 0.26	0.071
HI	0.06 ± 0.00	0.06 ± 0.00	0.343
D_2%_(Gy)	47.46 ± 0.19	47.50 ± 0.19	0.059
D_98%_(Gy)	44.53 ± 0.06	44.53 ± 0.05	0.703
D_mean_(Gy)	46.33 ± 0.07	46.34 ± 0.08	0.212

### Dosimetry comparison of OARs

**Fig. 4:**
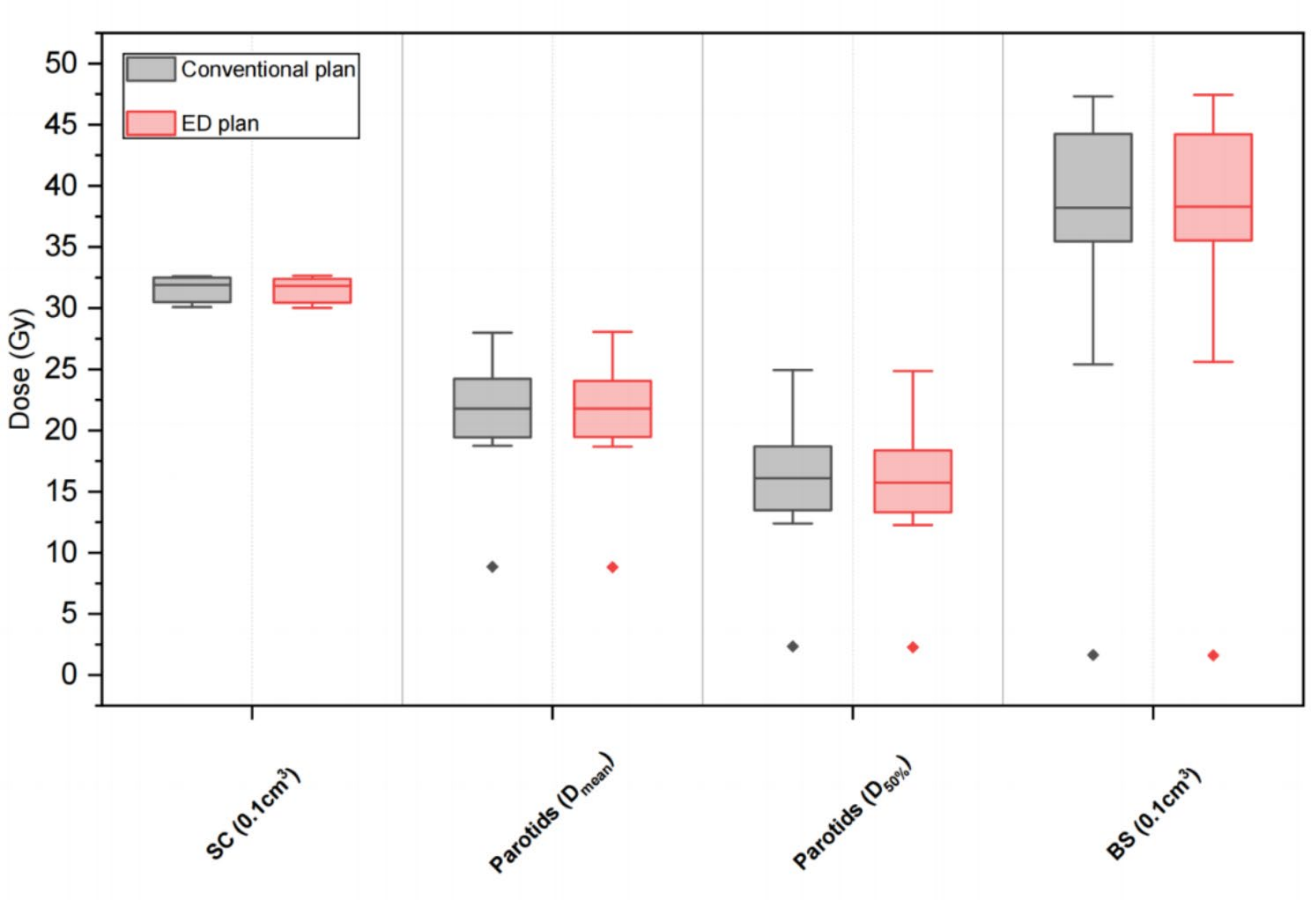
Box plots comparing key DVH parameters of OARs for conventional plans and ED plans in the head & neck treatment site. SC: spinal cord, BS: brain stem

**Fig. 5:**
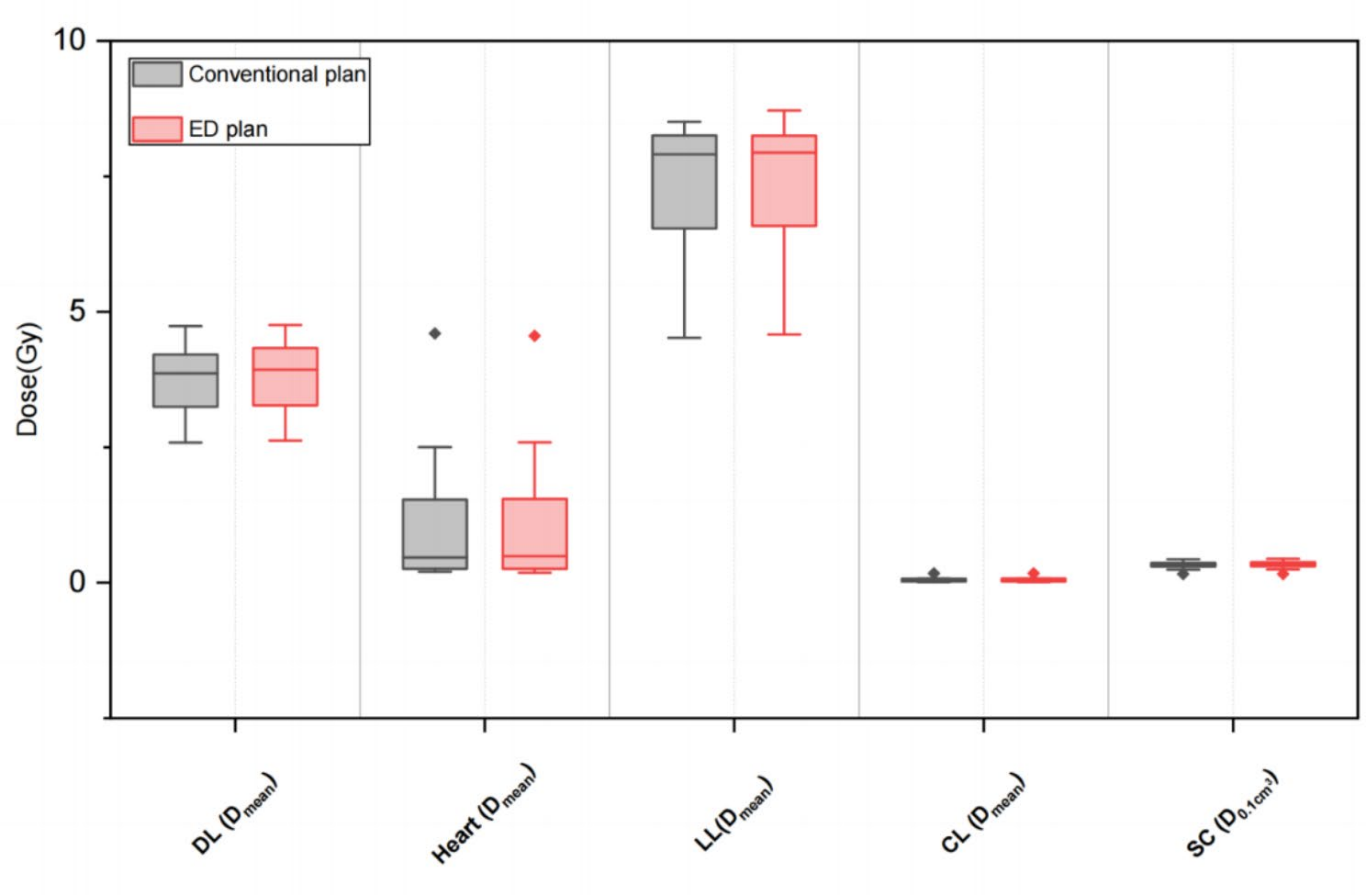
Box plots comparing key DVH parameters of OARs for conventional plans and ED plans in the chest treatment site. DL: Double lung, LL: Lateral lung, CL: Contralateral lung, SC: Spinal cord

**Fig. 6:**
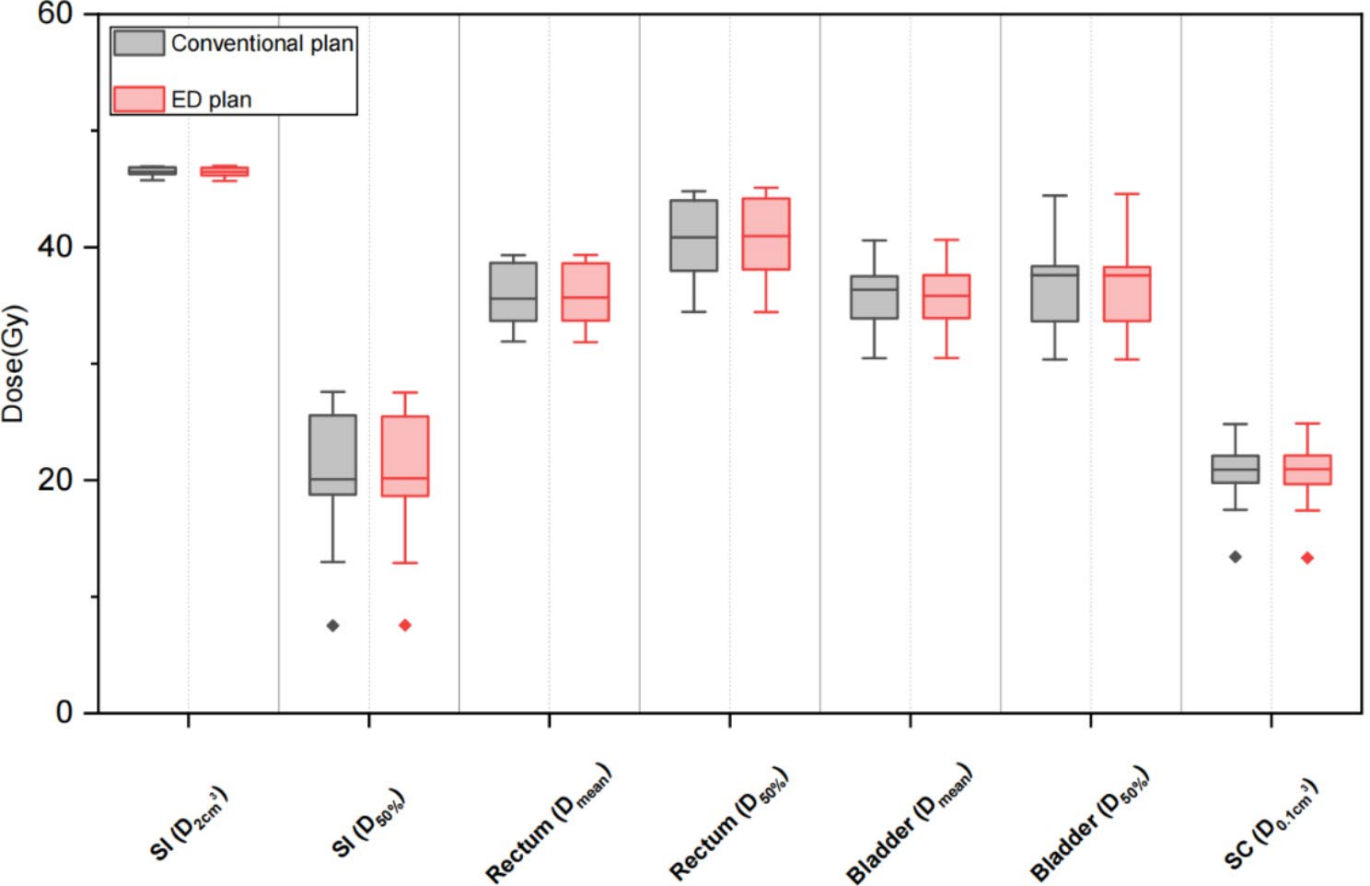
Box plots comparing key DVH parameters of OARs for conventional plans and ED plans in the pelvis treatment site. SI: Small intestine, SC: Spinal cord

The dosimetric differences in several key OARs between conventional plans and ED plans are listed in Table 5 and visualized as boxplots in Figs. 4, 5, and 6 for 3 treatment sites. In general, the statistical differences in DVH parameters between these two plans were observed mainly in the head & neck treatment site, but the differences in DVH parameters’ values remain minor. The maximum relative difference was only 1.29% without clinical significance. In addition, a similar situation was observed for the rectum D_50%_ in the pelvis treatment site. Besides, no clinically and statistically significant dosimetric differences were observed. The gamma analysis results for the two types of treatment plans were listed in Table 6.

**Table 5:** DVH parameters comparison of conventional plans and ED plans for key OARs (Mean ± SD)

OAR	DVH parameters	Conventional plans	ED plans	*P* value
**Head&Neck**				
Spinal cord	${\text{D}}_{{\text{0.1}\text{cm}}^{\text{3}}}$ (Gy)	31.53 ± 1.06	31.49 ± 1.41	0.403
Parotids	D_mean_ (Gy)	21.15 ± 5.42	20.09 ± 5.40	0.086
	D_50%_ (Gy)	15.45 ± 5.80	15.25 ± 5.75	0.002
Brain stem	${\text{D}}_{{\text{0.1}\text{cm}}^{\text{3}}}$(Gy)	35.64 ± 13.60	35.77 ± 13.64	0.008
**Chest**				
Double Lung	V_20Gy_ (%)	7.52 ± 1.90	7.53 ± 1.94	0.818
	D_mean_ (Gy)	3.73 ± 0.72	3.76 ± 0.73	0.114
Lateral lung	D_mean_ (Gy)	6.63 ± 2.68	6.67 ± 2.70	0.205
Contralateral lung	D_mean_ (Gy)	0.04 ± 0.03	0.04 ± 0.03	0.089
Heart	V_5Gy_ (%)	2.93 ± 5.23	2.97 ± 5.27	0.199
	D_mean_ (Gy)	1.17 ± 1.41	1.18 ± 1.42	0.229
Spinal cord	${\text{D}}_{{\text{0.1}\text{cm}}^{\text{3}}}$ (Gy)	0.32 ± 0.08	0.33 ± 0.08	0.000
**Pelvis**				
Small intestine	${\text{D}}_{{\text{2}\text{cm}}^{\text{3}}}$ (Gy)	46.50 ± 0.38	46.47 ± 0.41	0.222
	D_50%_ (Gy)	20.07 ± 6.20	20.03 ± 6.17	0.106
Rectum	D_mean_ (Gy)	35.95 ± 2.56	36.00 ± 2.59	0.085
	D_50%_ (Gy)	40.45 ± 3.65	40.55 ± 3.72	0.014
Bladder	D_mean_ (Gy)	35.81 ± 3.10	35.75 ± 3.11	0.607
	D_50%_ (Gy)	36.73 ± 4.15	36.75 ± 4.20	0.306
Spinal cord	${\text{D}}_{{\text{0.1}\text{cm}}^{\text{3}}}$ (Gy)	20.48 ± 3.18	20.48 ± 3.25	0.989

**Table 6:** Summary of gamma analysis results of conventional plans and ED plans

GPRs	1%/1 mm (%)	2%/2 mm (%)	3%/2 mm (%)
Head&Neck	91.02 ± 8.32	99.25 ± 0.68	99.59 ± 0.35
Chest	94.54 ± 9.65	99.75 ± 0.51	99.80 ± 0.47
Pelvis	96.89 ± 2.70	99.57 ± 0.24	99.71 ± 0.20

## Discussion

In the diagnostic X-ray energy range, X-ray attenuation is mainly composed of the photoelectric effect and Compton effect [17]. The attenuation of any material without a measurable K edge can be modeled as a combination of the photoelectric effect and the Compton effect. Because these interactions depend on Z_eff_ and ED, the Z_eff_ and ED value can be obtained by energy analytical solution [6]. Previous studies have validated the accuracy of determining ED values using dual-layer spectral CT systems, and the accuracy is not sensitive to the change of scan and reconstruction settings. Our test based on the clinical simulation scanning protocols also showed acceptable performance. The largest percentage deviations were observed in lung inserts (lung inhale and lunge exhale), which also appeared in the other studies [8, 18]. Based on this, we conducted further research to utilize ED maps generated by dual-layer spectral CT for dose calculation of treatment plans. In general, the gamma analysis results showed that the two dose distributions of conventional plans and ED plans were very similar. Even under the most stringent 1%/1 mm criterion, the lowest gamma passing rate was still higher than 90%. For the DVH parameters, there were no profound differences between conventional plans and ED plans. In terms of PTV, only the difference in CI of head&neck treatment site showed statistical significance, whereas the numerical difference is only 0.01 without practical significance. For OARs, the largest dose difference was 0.2 Gy found in the D_50%_ of parotids. From our experience, such dosimetric differences are unlikely to have clinical effects.

Since the dual-layer detector can acquire the two-level X-ray data at the same time, Philips spectral CT 7500 enables the simultaneous obtaining of conventional CT image data and spectral image data without selecting specific spectral scanning protocols. Using it to perform treatment plan transplantation and dose calculation can eliminate the errors derived from deformable registration between images. In our center, we have implemented accuracy determination of ED maps in the spectral CT quality control and quality assurance programs. Generally, we use the clinical simulation scanning protocols to scan the 062 M ED phantom, and ROIs of 75% of the rod diameter were selected to measure the inserts’ ED value of corresponding ED maps. Due to the treatment plans based on ED maps are not actually used in the clinics and there is a lack of guidelines and standards for ED measurement accuracy, our monthly quality control and quality assurance programs only record relevant measurement data for internal reference. Then, the measured and expected values are compared and analyzed. Currently, the community urgently needs to establish relevant guidelines and standards so that the ED map can be used in clinical settings.

In the radiation oncology community, spectral CT has gained increased interest due to its increased use in oncological radiology [19]. In spite of its potential to improve tumor visualization and characterization, most past studies focused on improving brachytherapy and proton therapy dose calculation due to the steeper dose gradient requiring more precise modeling for optimal therapy planning [20–22]. At the present stage, the community agrees that tissue attenuation can be estimated accurately to some extent when using HU information from conventional CT for megavoltage photons in external beam radiation therapy [23]. However, HU information must be converted into ED information by the HU-ED calibration curves and then can be applied to dose calculation. Therefore, medical physicists need to perform phantom calibration for specific simulation scanning protocols to acquire the data for developing corresponding HU-ED curves [24]. Once this work is completed, patients’ simulation scans are limited to using these protocols to avoid subsequent errors caused by deviations from the phantom calibration settings. This workflow does not ensure that individual patients can get the optimal CT images. The use of ED maps for dose calculation can eliminate the need for the HU-ED calibration curves and overcome the limitation. Hence, medical physicists no longer need to establish the HU-ED calibration curves and perform tedious quality assurance work for them. In addition, most commercial TPSs require manual selection of HU-ED calibration curves for CT images. Using ED maps for dose calculation can eliminate the risk of selecting the HU-ED calibration curve incorrectly, especially for large centers with multiple CT simulators.

The ED maps combined with other homologous spectral images will break through the traditional sketch of treatment planning. One of the foremost advantages of spectral CT in radiotherapy lies in its capacity to discriminate between different tissue types with greater clarity than conventional CT imaging. This enhanced tissue characterization facilitates more accurate delineation of target volumes and critical OARs, leading to improved treatment planning and dose calculation accuracy [7, 19, 23]. Metallic implants present a significant challenge in radiotherapy planning due to their pronounced artifacts on conventional CT images, which can distort dose calculations and compromise treatment accuracy. Spectral CT’s ability to mitigate metal artifacts through material decomposition techniques, as elucidated by wang et al [25]. and Zhao et al. [26], holds immense promise for optimizing dose calculation in the presence of metallic implants. By accurately delineating the extent of metal-induced artifacts and their impact on dose distribution, clinicians and medical physicists can devise more effective treatment plans while minimizing the risk of radiation-induced toxicities. Future research should validate the accuracy of the dose calculation using the ED map for treatment plans with high gradients, such as stereotactic body radiation therapy (SBRT), which usually requires smaller dose calculation grids and higher dose calculation accuracy.

In conclusion, we demonstrated the feasibility of using the ED map for dose calculation with existing commercial TPS. Compared with conventional treatment plans based on CT images, the treatment plans based on ED maps had similar dosimetric quality. However, the latter can be directly used in dose calculation without establishing and selecting any specific HU-ED calibration curve, providing an advantage in clinical applications.

## Data Availability

No datasets were generated or analysed during the current study.

## Abbreviations

CT: computed tomography
ED: Electron density
DECT: Dual-energy CT
VMI: virtual monochromatic images
VNC: virtual non-contrast
Zeff: effective atomic numbers
AAA: Anisotropic analytic algorithm
PO: phonon optimizer
FF-IMRT: fixed-field intensity modulated radiation therapy
VMAT: volumetric modulated arc therapy
PTV: Planning target volume
OARs: Organs at risk
FOV: Field of view
ROIs: Regions of interest
GPRs: gamma passing rates
HU: Hounsfield unit
CI: Conformity index
GI: Gradient index
HI: Homogeneity index
SBRT: stereotactic body radiation therapy
